# The Association between Hyperactivity and Suicidal Behavior and Attempts among Children Referred from Emergency Departments

**DOI:** 10.3390/ejihpe14100172

**Published:** 2024-09-24

**Authors:** Oren Shahnovsky, Alan Apter, Shira Barzilay

**Affiliations:** 1 Department of Community Mental Health, Faculty of Social Welfare and Health Sciences, University of Haifa, Haifa 3103301, Israel; oshahnov@campus.haifa.ac.il; 2 Department of Psychiatry, Schneider Children’s Medical Center of Israel, Faculty of Medicine, Tel Aviv University, Tel Aviv 4920235, Israel; eapter@clalit.org.il; 3 Faculty of Psychology, Reichman University, Herzliya 46150, Israel

**Keywords:** ADHD, children suicide, hyperactivity, self-report, parent report

## Abstract

The global prevalence of suicidal behaviors in children is rising, with attention-deficit hyperactivity disorder (ADHD) proposed as a contributing factor. This study examines the association between ADHD facets (hyperactivity and inattention) and suicidal behavior and attempts in children. Additionally, it seeks to compare self-reported ADHD symptoms and suicide-related incidents with parental reports. A cohort of 71 children referred from emergency departments due to suicidal thoughts and behaviors completed self- and parental report questionnaires. The results revealed that elevated hyperactivity scores, surpassing the ADHD diagnosis threshold, were significantly associated with increased rates of suicidal behavior. Hyperactivity demonstrated a stronger association with lifetime suicide attempts compared to inattention. Moreover, children’s self-reported ADHD symptoms exhibited a stronger correlation with suicide attempts than parental reports. This study highlights the critical role of hyperactivity in understanding suicidal behaviors among children with ADHD. It underscores the importance of considering hyperactivity-related symptoms in assessment and treatment approaches for suicidal behavior in this population.

## 1. Introduction

Suicide is the second leading cause of death among youth aged 10–18 [1]. Over the past decade, instances of non-fatal suicidal behavior have increased among preadolescent children [2]. This trend is evident in the growing number of children being brought to psychiatric and pediatric emergency rooms for suicide risk assessments [3], placing a significant strain on these healthcare services.

Although the issue is becoming more prevalent, research has primarily focused on suicidal risk among teenagers, with less emphasis placed on children under 12. This is partly due to the rarity of suicide attempts in this age group, as suicidal ideation is more common among adolescents [4]. Additionally, children exhibit clinical differences from their adolescent peers, underscoring the need for individualized evaluation and treatment strategies [5]. Moreover, discrepancies in assessing suicidal ideation can arise from parents’ lack of awareness or the child’s denial of such thoughts, which are more prevalent in younger children and decrease with age [6,7]. These discrepancies can complicate the recognition of suicidal risk in prepubertal children.

Nevertheless, research on suicide risk among children has gained increasing attention in recent years. Non-fetal suicidal behaviors have become relatively common among both clinical and community populations of children. A systematic review and meta-analysis conducted by Liu, et al. [8] found that the lifetime prevalence rates of suicidal ideation, suicide attempts, and non-suicidal self-injury (NSSI) among children were found to be 15.1%, 2.6%, and 6.2%, respectively. Studies have also identified several risk factors associated with suicidal behavior, including depression [4], anxiety and conduct symptoms [9], a family history of mental disorders and suicide, and parent–child conflict [10].

Research consistently shows that children with attention-deficit hyperactivity disorder (ADHD) are at a significant risk for experiencing suicidal thoughts and behaviors [8]. Preadolescents who attempt to hurt themselves often have ADHD, either comorbid with other mental health disorders or as the sole diagnosis, and, sometimes, it is more prevalent than depression [11]. Furthermore, children diagnosed with ADHD often face severe difficulties and are at a higher risk of attempting suicide or engaging in NSSI even ten years later [12]. However, it remains unclear which specific aspects of ADHD are linked to suicidal behaviors in children.

The original conceptualization of ADHD, proposed by Barkley [13], suggested that the disorder involved both impulsivity and hyperactivity. Under the current diagnostic criteria, these two symptoms are combined, as the emphasis on specific symptoms can be inconsistent and may vary over time [14]. Hyperactivity is characterized by restlessness, constant talking, fidgeting, and difficulty staying focused on a task [15], and it is often associated with risky decision making [16]. Excessive hyperactivity in children can have lasting negative effects on both the child and their family, leading to significant declines in overall functioning, aggressive behaviors, mood and anxiety disorders, and inappropriate coping strategies that interfere with academic performance and social functioning [17].

Hyperactivity is a common presentation that can also be manifested across various settings and can be reported by multiple informants, such as at home and school. However, there is often low agreement between children and their informants [18]. Research on informant disagreement in childhood ADHD has shown that parents often underestimate their children’s symptoms compared to teachers [19]. The levels of agreement between informants are influenced by various factors, including the child’s individual characteristics. Supporting this notion are findings that suggest informant–patient agreement is higher in nonclinical samples than in clinical samples. Moreover, while patients tend to underreport hyperactivity symptoms, they are still considered the best informants regarding their symptoms [20].

It is now well established that significant associations exist between ADHD and suicidal behavior and that stimulant medications may help attenuate this link [21]. Research suggests that individuals with the combined type of ADHD may have a higher risk for suicide attempts, particularly when inattention is a prominent component of the disorder [22]. Other studies have indicated that the increased levels of hyperactivity and impulsiveness observed in people with ADHD could be the primary factors connecting the disorder to suicidal behavior [23]. Indeed, several studies have found impulsivity to be a crucial predictor of suicidal tendencies in individuals with ADHD, showing a strong positive correlation between the two variables [24].

It is important to note that the impact of hyperactivity symptoms on suicide risk can vary among individuals, influenced by contextual factors such as social and cultural contexts, which have long been recognized as crucial in understanding the phenomenon of suicide [25,26,27]. Substantial evidence suggests that ADHD can lead to social and relational difficulties, which may, in turn, exacerbate the risk of suicidal thoughts and behaviors. The influential role of social factors was first highlighted by Émile Durkheim, who suggested that social and moral integrations are associated with increased suicide rates [28]. Building on this foundational work, the Interpersonal Theory of Suicide (IPTS), proposed by Joiner [29], posits that two interpersonal cognitions—thwarted belongingness and perceived burdensomeness—drive the inclination toward suicide, with both exacerbated by a sense of hopelessness. IPTS assumptions have been supported in predicting suicidal thoughts and behaviors among adolescents [30,31]. Additionally, findings suggest that various aspects of social contexts, including parental affection, peer support, and school environments, significantly influence changes in suicide ideation and the risk of suicide attempts over time [32,33]. Social support has also been identified as a mediator between ADHD symptoms and emotional outcomes, including suicidal considerations in young adulthood [34]. These insights underscore the necessity of considering social and relational factors when examining the relationship between ADHD symptoms and suicide risk, as these factors may play a pivotal role in shaping the development of suicidal thoughts and behaviors in this population.

This study builds on prior research exploring the link between ADHD and suicidal behavior in children, with a focus on the systems of hyperactivity and inattention. The aim is to uncover the distinct connections these symptoms have to suicidal behaviors and attempts, while also considering variations in symptom ratings from different informants. We hypothesize the following:

**H1:**  *Children referred from the emergency department who exhibit ADHD symptoms above clinical thresholds are more likely to engage in suicidal behavior and attempts*.

**H2:**  *While ADHD is generally associated with these outcomes, only hyperactivity (not inattention) will show a significant correlation*.

**H3:**  *Given the discrepancies in hyperactivity reports from different sources, we anticipate differences in associations between self-reports by children and their parents’ reports concerning hyperactivity symptoms and suicidal behavior*.

## 2. Materials and Methods

### 2.1. Participants

Seventy-one children, referred from the emergency department and admitted to the depression and suicide clinic at a university-affiliated children’s hospital, were included in this study [35]. Enrollment took place from May 2018 until November 2021. The inclusion criteria were ages 7–12 years, and a history of suicidal ideation, behavior, or attempt. Exclusion criteria included developmental or neurological disorders and intellectual disabilities that would prevent the understanding of research questions, as determined by a licensed clinician (psychiatrist or clinical psychologist).

### 2.2. Procedure

The study procedures were approved by the Institutional Review Board of the Medical Center. During their initial clinic visit, 1–3 weeks after being referred from the emergency department, senior child psychiatrists and clinical psychologists briefed participants on the study elements and processes. After receiving approval, the participant’s parent(s) provided informed consent, allowing their questionnaire data to be used. Trained research assistants guided participants in completing questionnaires on individualized computers via REDCap electronic tools [36], a process that took approximately 30 min. Based on the clinical evaluation, participants were recommended pharmacological or psychological interventions following the intake session. Previous research suggests that asking questions about suicidal thoughts and behaviors does not induce additional distress [37]. To further mitigate the risks of discomfort, research staff were available during the assessment to assist in the case that participating children and parents might have found certain questions boring, difficult, or uncomfortable. Additionally, during the informed consent process (and reiterated during the research assessments), the participants were told they were not obliged to answer any questions and could withdraw from the study at any time without consequences.

### 2.3. Measures

#### 2.3.1. Socio-Demographic Characteristics

Parents were asked to provide socio-demographic details, as regional and demographic patterns might contribute to the variation in suicide rates, particularly in children [9]. Specifically, we asked about their family status, profession, education, and socioeconomic status, based on findings from a nationwide survey of adolescents in Israel that linked increased risks of suicide attempts to factors such as parental unemployment and involvement in welfare programs [38]. We also inquired about immigration status, given that higher suicide rates have been observed among immigrants from the former Soviet Union and Ethiopia in Israel [39].

#### 2.3.2. Suicide Ideation and Behavior

To assess suicidal ideation and behavior, we used the screening version of the Columbia Suicide Severity Scale (CSSRS) [40], a validated measurement tool consisting of six dichotomous self-report items (Yes/No). Items 1–5 assess suicidal thoughts, intentions, and planning over the past two weeks, and item 6 assesses a lifetime history of suicidal behavior. Participants who answered “yes” to at least one of the ideation items were categorized as having suicidal ideation. The CSSRS also includes an item assessing the lifetime incidence of NSSI. Lifetime suicidal behavior was determined by the score on item 6, which asks about specific suicidal behaviors (“Have you ever done anything, started to do anything, or prepared to do anything to end your life?”). An additional item was included to assess suicide attempts, asking participants if they had ever attempted to kill themselves (“Did you do anything to try to kill yourself or make yourself not alive anymore?”). The CSSRS has demonstrated good psychometric characteristics [41]. The internal consistency of the CSSRS in our sample is high (α = 0.95).

#### 2.3.3. Strengths and Difficulties Questionnaire (SDQ)

Goodman [42] developed the Strengths and Difficulties Questionnaire (SDQ) to evaluate emotional and behavioral issues in children and adolescents. The SDQ consists of 25 items, divided into five subscales: Emotional Symptoms, Conduct Problems, Hyperactivity/Inattention Problems, Peer Relationship Problems, and Pro-Social Behavior. Each item is rated on a 3-point scale from 0 (“not true”) to 2 (“certainly true”), with a maximum score of 10 for each subscale.

Previous research examined the efficacy of the SDQ in identifying ADHD [43]. The hyperactivity–inattention subscale has been found to yield accurate results in self-reported data, making it a useful tool for diagnosing ADHD when other diagnostic options are unavailable, as was the case in this study. While we considered relying on participants’ medical history to identify ADHD, it was under-reported. Diagnosing ADHD typically requires information from multiple informants, such as parents, teachers, and clinicians [14], which is also not feasible in an emergency department setting. Additionally, the diagnosis often involves standardized tests that are also unavailable [44]. Therefore, we used the SDQ as a validated instrument to identify ADHD symptoms.

Elevated scores on the subscales, as reported by parents, have been shown to increase the likelihood of meeting ADHD criteria, making it a valid tool for differentiating between ADHD and non-ADHD cases [45]. Therefore, we utilized the hyperactivity–inattention subscale of SDQ to determine ADHD diagnosis, as reported by both the participants and their parents. This subscale was further divided into two separate factors: the first factor comprised items 2 (“restless”) and 10 (“fidgeting”), which loaded onto the hyperactivity factor; while the second factor included items 15 (“distracted”), 21 (“thinks things out”), and 25 (“attention span”), which loaded onto the attention deficit factor [43]. A cut-off score of 6 or higher for self-reports and 5 or higher for parent reports was used to validate the existence of ADHD as a dichotomous variable (yes/no) in a previous epidemiological study [43]. The internal consistencies of the SDQ scale and subscales were adequate in our sample (Cronbach’s alpha α = 0.67–0.70 for children’s and parents’ reports, respectively).

### 2.4. Data Analysis

The statistical analysis was conducted using SPSS software, version 27.0 for Windows. An a-priory power analysis using G*Power 3 [46] was conducted before data organization and analysis, revealing that a minimum number of 86 participants should be sufficient for achieving a power of 0.8- for a medium effect size d = 0.50 and an alpha of 0.05. After data cleaning, 71 participants with completed data were included in the data analysis. Next, variables were reviewed for data entry accuracy and missing values, with missing data managed assuming randomness. The Missing Values Analysis indicated no significant departure from Missing Completely at Random (MCAR) based on Little’s test (*χ*^2^_(92)_ = 108.472, df = 92, *p* = 0.11), affirming MCAR assumption. Multiple Imputation with 20 imputed datasets was conducted [47], and a conservative selection of 6 burn-in iterations was chosen based on imputation convergence assessment. No significant disparities were observed between those who completed the questionnaire and those who did not. Subsequently, a chi-square test for independence examined links between recent suicidal behavior, lifetime suicide attempt, and hyperactivity–inattention (above the clinical threshold), reported separately by children and parents. Further exploration utilized logistic multivariable regression to probe the relationship between hyperactivity and inattention factors and suicide attempts. Age and gender were included as covariates in the logistic regression analysis. The enter method was applied, involving all predictor variables concurrently in the regression model, irrespective of their statistical significance.

## 3. Results

### 3.1. Descriptive Statistics

This study included 71 children aged 7–12 (M = 10.48, SD = 1.38). Among them, 33 were males (46.5%), 37 were females (52.1%), and 1 identified otherwise (1.4%). Of the participants, 22.5% demonstrated recent suicidal behavior, 31% had a lifetime suicide attempt, 47.9% had recent suicidal ideation, and 38% had a history of NSSI. We also investigated hyperactivity, noting discrepancies between informants [18]—31% met the threshold via self-reports, 33.8% via parental reports, and 16.9% via both. See Table 1 for complete sample data.

### 3.2. Correlations between Hyperactivity and Inattention Symptoms and Suicide Attempt

To test the hypothesis that children with suicidal behavior and suicide attempts (dependent variables measured separately by C-SSRS binary scores) have higher proportions of clinical hyperactivity–inattention (independent variable, measured by the SDQ subscale above the cut-off binary score), we used the chi-square test of independence.

Consistent with our hypothesis, the analysis revealed a significant relationship between lifetime suicide attempts and self-reported hyperactivity–inattention (*χ*^2^_(1,71)_ = 5.38, *p* = 0.020, Cramer’s V = 0.276). This indicates that among children who attempted suicide, the proportion of clinical hyperactivity–inattention presence is higher. By contrast, no significant association was found between parents’ reports of hyperactivity–inattention and lifetime suicide attempts, which did not support our hypothesis.

### 3.3. Suicidal Behavior

An additional chi-square test of independence, examining the relationship between self-reported recent suicidal behavior and hyperactivity–inattention, revealed that this relationship approached significance for self-reports. That, partially supports our hypothesis, indicating a potential link between suicidal behavior and clinical hyperactivity–inattention presence (*χ*^2^_(1,71)_ = 3.49, *p* = 0.062, Cramer’s V = 0.222). However, contrary to our hypothesis, no significant relationships were found between suicidal behavior and clinical hyperactivity–inattention as reported by parents. All chi-square correlations are presented in Table 2 below.

Due to the discrepancies between the self-reports and parental reports, we evaluated the agreement between ADHD self-reports and parental reports using Kendall’s W coefficient of concordance. The results indicated a very low level of agreement (Kendall’s W = 0.003, *χ*^2^ = 0.182, *p* = 0.67), suggesting almost no concordance between the children’s and parents’ reports on ADHD symptoms.

### 3.4. Hyperactivity vs. Inattention Symptoms and Suicidality

Given the significant relationship found between lifetime suicide attempts and self-reported hyperactivity–inattention presence, we further investigated the link between these variables using logistic multivariable regression. Logistic regression was selected for its ability to manage multiple independent variables and assess their unique and combined effect on a binary outcome. Specifically, this approach allowed us to concurrently examine the prediction of lifetime suicide attempts by hyperactivity and inattention symptoms. We also included age and gender as covariates in the logistic regression analysis. We hypothesized that hyperactivity would be correlated with lifetime suicide attempts rather than inattention.

The logistic regression results displayed in Table 3 highlight the relationship between self-reported hyperactivity and inattention and suicide attempts. Consistent with our hypothesis, we found a significant association between hyperactivity and suicide attempts (Exp_(B)_ = 1.922, 95% CI = 1.061–3.483, *p* = 0.031), whereas no significant association was observed between inattention and suicide attempts. The model’s explanatory power was evaluated using Cox and Snell *R*^2^, found to be 0.166. This indicates that approximately 16.6% of the variance in lifetime suicide attempts is explained by the model, highlighting a moderate fit of the predictors to the outcome variable. Furthermore, no significant associations were observed when investigating the link between suicidal behavior and self-reported hyperactivity and inattention. Lastly, age and gender were not significantly associated with suicidal behavior and attempt.

## 4. Discussion

The main findings of this research underscore a significant association between self-reported suicidal behaviors and hyperactivity among children referred from the emergency department. Differences between child and parental reports on suicidality and hyperactivity were also observed. Notably, hyperactivity appears to be more closely linked to self-reported suicidal behavior in children than inattention, although this relationship should be interpreted with caution and warrants further investigation. This suggests the importance of specifically examining hyperactivity in suicide risk assessments, rather than focusing solely on inattention. While suicide attempts have many other potential predictors, such as depression and anxiety [9], this study was designed to specifically investigate the association between ADHD symptoms and suicidal behaviors in children.

Building on previous research that identified low child–caregiver concordance between child and caregiver reports of suicidality [7], our study aimed to investigate whether discrepancies existed between children’s self-reports of hyperactivity symptoms and their parents’ reports, particularly in relation to suicidal behavior and attempts. Our findings support the hypothesis that children’s self-reports may serve as a more accurate indicator of suicidal behavior than parental reports.

Informant discrepancies are a prevalent issue in reporting a child’s symptoms across various mental health domains, including the assessment of suicidal thoughts. Parental unawareness and patients’ denial of suicidal thoughts are common and often result in significant disagreements among reporters when evaluating risk. Age also plays a crucial role, as agreement between self-reported and parent-reported symptoms is generally low in younger children compared to adolescents [6]. Similar discrepancies arise when assessing attention and hyperactivity [48].

Numerous studies have explored the factors affecting consensus between various sources when reporting symptoms. These studies consistently show that parent and child ratings of externalizing symptoms, such as aggression, tend to exhibit higher levels of agreement compared to internalizing symptoms, such as depression [49]. Additionally, children may have a unique advantage over their parents in observing certain mental health domains, such as anxiety and behaviors exhibited during peer interactions [48].

This study indicated that children tend to be more reliable in reporting their hyperactivity, particularly when considering its various impacts. While parents may focus primarily on the symptoms and their own distress, children also report related consequences on their peer relationships and internalizing symptoms, providing a more comprehensive perspective. This expanded viewpoint may lead to reduced agreement among informants, as observed in our findings and supported by prior research [48].

### 4.1. Implications

The current findings suggest the importance of incorporating specific evaluations of hyperactivity symptoms, alongside inattention, when conducting suicide risk assessments for children under the age of 12. While ADHD may be a significant risk factor, it is important to screen for relevant symptoms cautiously. This study also indicates notable discrepancies between children’s and their parents’ reports, particularly concerning hyperactivity and suicidal behaviors. Therefore, clinicians are suggested to employ a multi-informant assessment approach that includes reports from both children and parents, as these may provide a more comprehensive understanding of the child’s mental health. Additionally, educating parents on how to recognize their child’s symptoms and potential warning signs of suicidal thoughts and behaviors remains crucial.

### 4.2. Limitations

The majority of clinical outcomes relied on self-reported data from children, which often raises concerns about the validity of child report instruments and adds complexity to result interpretation [50]. Additionally, a key limitation of this study is its relatively small sample size, which may affect the power and generalizability of our findings. Future research should aim to include a larger sample to ensure more robust and representative results. While the current ADHD diagnosis was based on a reliable and well-known questionnaire, it is important to note that the absence of the patient’s medical history and input from multiple informants beyond caregivers, such as teachers, limits the comprehensiveness of the ADHD assessment. Future research should adopt a more holistic approach by including the patient’s full medical history and gathering input from various informants, such as teachers, to ensure a more accurate and contextually rich diagnosis of ADHD. Another potential limitation of this study is the influence of the parent’s emotional and mental state on their responses. The stress and anxiety associated with an emergency department visit due to a child’s risk for suicide may have affected the parents’ perceptions and reporting. This might also contribute to the discrepancies between parent and child reports, as found in previous studies [7]. Moreover, the link between ADHD and lifetime suicide attempts found in this study may be influenced by psychiatric comorbidities, such as depression, although this study primarily focused on ADHD’s distinct role. Furthermore, we did not account for whether the participants were taking medications, despite evidence that antidepressants can have side effects that significantly increase the risk of suicidal ideation and attempts among young people [51]. Those factors were considered in a separate study [9] and warrant further investigation in future research. Lastly, this study’s cross-sectional design prohibited the establishment of causation or temporal sequence among the variables studied.

### 4.3. Future Research

Examining the link between ADHD symptoms and children’s suicidal behavior is pivotal for effective prevention and intervention strategies. Although our study centered on hyperactivity as an ADHD indicator, it is vital to explore other ADHD symptoms concerning child suicidality. Impulsivity, often linked with hyperactivity, is extensively studied as a suicidal behavior risk factor [24]. For deeper understanding, future research should employ robust impulsivity measures like the UPPS Impulsive Behavior Scale, assessing various impulsive behavior aspects [52]. Comprehensive ADHD diagnosis should involve diagnostic interviews and multiple sources (children, parents, teachers). While this current study focuses on the children and their parents, there is evidence to suggest that ADHD generates social and relational problems that can lead to suicidal behaviors. Therefore, future research should incorporate a broader range of social factors to provide a more comprehensive understanding of the multifaceted nature of suicide risk among children. It should also explore the pathways through which ADHD leads to suicidal thoughts and behaviors and investigate the unique role of potential mediators such as interpersonal difficulties, impulsivity, and emotional dysregulation.

## 5. Conclusions

This study contributes to the limited research on suicidality among children under the age of 12 by exploring the potential link between hyperactivity, as a symptom of ADHD, and suicidal behaviors and attempts. The results highlight the importance of evaluating hyperactivity symptoms during clinical and risk assessments of young children referred from emergency departments. While the findings suggest that hyperactivity may be more critically correlated with suicidal behavior among children than inattention, it is important to approach these results with caution. This emphasizes the need to consider various aspects of ADHD when identifying risk factors for suicide, highlighting the significance of a comprehensive approach. Additionally, this study suggests that children’s self-reports of their hyperactivity symptoms may provide a more accurate assessment of their risk for suicide outcomes compared to reports provided by their parents. Therefore, incorporating input from multiple informants and carefully examining any discrepancies in their reports is crucial. By doing so, healthcare professionals and caregivers can develop more effective prevention and intervention strategies for children at risk of suicidal behavior, especially those referred from emergency departments.

## Figures and Tables

**Table 1:** Frequency distribution of ADHD, suicidal outcomes, socio-demographic characteristics, and descriptive statistics.

Variable				n	%
ADHD by Self-Report ^1^				22	31%
ADHD by Parental Report ^2^				24	33.8%
ADHD symptoms (Self- and Parental Report) ^3^				12	16.9%
Suicidal Behavior				16	22.5%
Suicide Attempt				22	31%
Suicide Ideation				34	47.9%
Non-Suicidal Self-Harm				27	38%
Gender	Female			37	52.1%
	Male			33	46.5%
	Other			1	1.4%
Parent Immigration	Frequency			27	38%
Patient Immigration	Frequency			18	25.4%
Socioeconomic Status	Above Avg.			28	39.4%
	Avg.			27	38%
Parent Employment	Employed			56	78.9%
Parent Education	School			20	28.2%
	Academic/Professional			51	71.8%
Child Lives with	One Parent			25	35.2%
	Both Parents			46	64.8%
Siblings	Has Siblings			27	38%
Descriptive Statistical Information					
Variable	Mean	Median	SD	Min	Max
SDQ Hyperactivity–Inattention—Self-Report ^4^	4.73	5	2.23	0	9
SDQ Hyperactivity–Inattention—Parental Report ^4^	4.77	4.54	2.62	0	10
SDQ Hyperactivity—Self-Report ^5^	1.57	2	1.24	0	4
SDQ Hyperactivity—Parental Report ^5^	1.5	1	1.51	0	4
SDQ Inattention—Self-Report ^6^	3.16	3	1.54	0	5
SDQ Inattention—Parental Report ^6^	3.28	3	1.83	0	6

^1^ SDQ hyperactivity–inattention subscale cut-off (Self-Report): indicates a score of 6 or above on the SDQ hyperactivity–inattention subscale. ^2^ SDQ hyperactivity–inattention subscale cut-off (Parental Report): indicates a score of 5 or above on the SDQ hyperactivity–inattention subscale. ^3^ SDQ hyperactivity–inattention combined reports: represents the combined scores from self- and parental reports using the SDQ hyperactivity/inattention subscale. ^4^ SDQ hyperactivity/inattention subscale: the subscale of the SDQ used to assess hyperactivity and inattention symptoms. ^5^ SDQ hyperactivity factor: a subscale of the SDQ used to assess hyperactivity. ^6^ SDQ inattention factor: a subscale of the SDQ used to assess inattention.

**Table 2:** H1: proportions of hyperactivity–inattention presence above cut-off among those with suicidal behavior and who made suicide attempts—self-report and parental report.

	Suicide Attempt (n = 22) ^1^				Suicidal Behavior (n = 16) ^2^			
	N (Yes)	% of Attempt	*χ* ^2^	*p*	N (Yes)	% of Behavior	*χ* ^2^	*p*
Self-Report ^3^ (n = 22)	13	59%	5.38	0.020	9	56%	3.49	0.062
Parental Report ^4^ (n = 24)	12	55%	0.719	0.396	8	50%	0.913	0.339

^1^ Suicide Attempt—lifetime suicide attempt. ^2^ Suicidal behavior—recent suicidal behavior in the last two weeks. ^3^ Self-report—hyperactivity–inattention presence in self-reports determined by SDQ subscale cut-off. ^4^ Parental report—hyperactivity–inattention presence in parental reports determined by SDQ subscale cut-off.

**Table 3:** H2: hyperactivity and inattention presence as predictors of suicide attempts and suicidal behavior.

						95% C.I. for EXP(B)		
	Predictor Variables	B	S.E	*p*	Exp_(B)_	Lower	Upper	Cox and Snell *R*^2^
Suicide Attempt	Age	0.081	0.281	0.773	1.084	0.625	1.881	0.166
	Gender	0.538	0.661	0.416	1.712	0.469	6.249	
	Hyperactivity ^1^	0.653	0.303	0.031	1.922	1.061	3.483	
	Inattention ^2^	0.327	0.265	0.218	1.387	0.825	2.333	
Suicidal Behavior	Age	0.587	0.332	0.077	1.799	0.939	3.447	0.149
	Gender	1.141	0.728	0.117	3.131	0.752	13.042	
	Hyperactivity ^1^	0.315	0.303	0.298	1.370	0.757	2.479	
	Inattention ^2^	0.032	0.241	0.895	1.032	0.644	1.656	

^1^ Self-report SDQ hyperactivity factor. ^2^ Self-report SDQ inattention factor.

## Data Availability

The data supporting the findings of this study are available on reasonable request from the corresponding author.
